# Supplementary material for: Continuous Bose–Einstein condensation
Source: Nature. 2022 Jun 8;606(7915):683–7. doi: 10.1038/s41586-022-04731-z (PMC9217748; doi:10.1038/s41586-022-04731-z)
Supplement: Supplementary file 1 — This file contains Supplementary Methods to measure the reservoir loading rate, to detect the BEC anisotropy after time of flight and to model the open dynamics of the BEC. It also contains a Supplementary Discussion of the model and its findings, as well as of the optical dipole trap loading and BEC creation dynamics. [file 41586_2022_4731_MOESM1_ESM.pdf]

nature portfolio

https://doi.org/10.1038/s41586-022-04731-z

Supplementary information

Continuous Bose–Einstein condensation

In the format provided by the authors and unedited

Continuous Bose-Einstein condensation

Chun-Chia Chen (陳俊嘉)[1] Rodrigo González Escudero[1] Jiří Minář[2,3]
Benjamin Pasquiou[1,3] Shayne Bennetts[1,3] and Florian Schreck[1,3,*]

[1] *Van der Waals-Zeeman Institute, Institute of Physics, University of Amsterdam, Science Park 904, 1098XH Amsterdam, The Netherlands*
[2] *Institute for Theoretical Physics, Institute of Physics, University of Amsterdam, Science Park 904, 1098XH Amsterdam, The Netherlands*
[3] *QuSoft, Science Park 123, 1098XG Amsterdam, The Netherlands*

(Dated: March 15, 2022)

Characterizing the reservoir loading

To estimate the flux Φ_{R} of atoms loaded into the reservoir we begin with all laser cooling and trapping beams on, except for the Zeeman slower beam. After some time, a steady state is reached, in which the guide is filled but not the reservoir. We then switch on the Zeeman slower beam, and observe the loading of the reservoir, see Extended Data Fig. 2. Using our fitting procedure for the absorption images (see Methods section "Characterizing the BEC and thermal cloud"), we estimate the growth in the number of atoms in various regions of the cloud. With the rough assumptions of a constant flux Φ_{R} and a one-body loss rate parameter Γ_{loss} originating for example from transfer of atoms to the dimple, the loading of the reservoir can be fitted by the exponential growth function $N_{\mathrm{R}}(t) = (1 - e^{-\Gamma_{\mathrm{loss}} t})\, \Phi_{\mathrm{R}}/\Gamma_{\mathrm{loss}}$. This function fits best for a flux $\Phi_{\mathrm{R}} = 1.4(2) \times 10^6$ atoms s^{-1}, see Extended Data Fig. 2. We also show the combined number of atoms loaded in the reservoir and "crossing" (guide-reservoir intersection) regions. We fit these data with a similar exponential growth function, and obtain a flux of $2.9(3) \times 10^6$ atoms s^{-1}. The relative uncertainty of the combined atom numbers is smaller than for the data set describing only the reservoir atom number. This is due to ambiguity between the "reservoir" and "crossing" regions in our fitting procedure.

Loading dynamics

The data of Extended Data Fig. 2 show several timescales at play in the system's evolution. First we see atoms populating the reservoir. About 500 ms later the dimple region begins to fill. Finally about 1 s after the start of the dimple loading we see a BEC begin to form.

These dynamics can be understood from the thermalization timescale and from the need to exceed the critical phase-space density to begin forming a BEC. We estimate the peak phase-space density evolution by $\rho = N_D \left(\frac{\hbar^3 \omega_{Dx} \omega_{Dy} \omega_{Dz}}{k_B^3 T_D^3} \right)$, where N_D is the thermal atom number in the dimple. However, this estimation is inaccurate because of the non-thermalized distribution function describing the atoms in the dimple (see section "Modeling of the BEC's open dynamics" below). We give it here as an indication of the phase-space density, which with this definition and for a thermalized sample should be greater than 1.2 in order to produce a BEC.

Once the critical phase-space density is exceeded BEC formation begins at a slow rate and then accelerates as more atoms condense. Indeed, the growth of a BEC or matter-wave is governed by Bose-stimulated scattering, which scales with the number of atoms occupying the ground state of the trap[60–63].

After about 3 s steady state is established. We probe the system for various hold times up to 5 min and find no indication that the BEC departs from steady state. This is exemplified in Extended Data Fig. 2 and Fig. 3b.

BEC anisotropy after time-of-flight

A Bose-Einstein condensate and a thermal gas expand very differently when released from a trap. A thermal gas expands isotropically, beginning with a shape reminiscent of the initial trap geometry, and asymptotically approaching an isotropic shape for long times of flight (TOF). By contrast, the expansion of a BEC released from an anisotropic trap remains anisotropic for long TOF, inverting its aspect ratio mid flight. This originates from the anisotropic release of mean-field energy, which reflects the trap anisotropy[64].

The transpose-anisotropy provides an elegant method by which to efficiently detect the presence of a CW BEC. The transpose-anisotropy of the density distribution n_{OD} is $n^s_{\mathrm{OD}}(y, z) - n^s_{\mathrm{OD}}(z, y)$, where the origin of the coordinate system is at the density maximum. n^s_{OD} is obtained from n_{OD} by adding the same n_{OD} distribution rotated by 180°. Transpose-anisotropies for short (0.1 ms) and long (18 ms) TOFs are shown in Extended Data Fig. ??. For 0.1 ms TOF, the density distribution shows a marked anisotropy as indicated by a strong cloverleaf pattern. This initial anisotropy is solely due to the action of the trap geometry on the density distribution of the thermal gas, as the size of a potential BEC is below our imaging resolution. However, for 18 ms TOF we see a difference between pictures for short (2.2 s) and long (3.0 s) hold time t_{hold}. For short t_{hold} the anisotropy is broad and simply a remnant of the initial cloud anisotropy. However for long t_{hold}, at which steady state is established, we see an additional, smaller cloverleaf pattern with opposite anisotropy around the center of the picture. Both the existence and the sign of this pattern are consistent with the expansion of a BEC from a dimple with our trap frequencies.

* ContinuousBEC@strontiumBEC.com

Modeling of the BEC's open dynamics
In order to describe the formation and properties of the continuous-wave Bose-Einstein condensate, we develop a model capable of fitting our experimental data, as shown for example by the dashed blue line in Fig. 3b. In the following we describe the model and its results.

Firstly, we lay out the model, which builds on the standard Boltzmann kinetic theory of Bose gases with additional dissipation to account for the open dynamics. As a main result of our derivation we arrive at the rate equation Eq. (10). We provide details of the derivation of each gain and loss term in the equation and the assumptions that led to them.

Secondly, we discuss the fit of this model to our data, from which we extract useful physical quantities, like the 1- and 3-body loss rate parameters, and the flux into the CW BEC after reaching steady state.

In particular, we find that we are not able to describe our data if we assume that our system is in thermal equilibrium. This reflects the intrinsic driven-dissipative nature of the system.

The model — We model the dynamics of the BEC using the rate equation

$$\begin{aligned}\dot{n}_{\mathrm{BEC}}(\mathbf{r}) = s_{\mathrm{in}}(\mathbf{r}) - s_{\mathrm{out}}(\mathbf{r}) - \gamma_{1\mathrm{b}} n_{\mathrm{BEC}}(\mathbf{r}) \\ -\gamma_{3\mathrm{b}}\left[n_{\mathrm{BEC}}(\mathbf{r})^3 + 6 n_{\mathrm{BEC}}(\mathbf{r})^2 n_{\mathrm{th}}(\mathbf{r}) \right. \\ \left. +6 n_{\mathrm{BEC}}(\mathbf{r}) n_{\mathrm{th}}(\mathbf{r})^2\right],\end{aligned} \tag{1}$$

where $\gamma_{1\mathrm{b}}, \gamma_{3\mathrm{b}}$ are the phenomenological one- and three-body loss rate parameters, see[65], $n_{\mathrm{BEC}}, n_{\mathrm{th}}$ are the local densities of the BEC and thermal atoms in the dimple, and we do not write explicitly the time dependence of the variables. In the framework of kinetic Boltzmann equations, $s_{\mathrm{in}}, s_{\mathrm{out}}$ are the collisional integrals (source terms) describing the exchange between the thermal atoms and the BEC. To this end we follow closely the treatment in[66] and write (setting $\mathbf{v}_{\mathrm{BEC}} = 0$ in[66])

$$s_{\mathrm{in}} = \frac{n_{\mathrm{BEC}}\sigma}{\pi h^3}\int \mathrm{d}\mathbf{p}_3 f_3 \frac{1}{v_3}\int \mathrm{d}\tilde{\mathbf{v}} f_4 \tag{2a}$$

$$s_{\mathrm{out}} = \frac{n_{\mathrm{BEC}}\sigma}{h^3}\int \mathrm{d}\mathbf{p}_2 f_2 \Delta v \int \frac{\mathrm{d}\Omega}{4\pi}(1+f_3+f_4), \tag{2b}$$

where $\sigma = 8\pi a_{\mathrm{sc}}^2$ is the s-wave scattering cross-section, $\Delta v = \sqrt{v_2^2 - 4v_0^2}$, $v_0 = \sqrt{g n_{\mathrm{BEC}}/m}$, $g = 4\pi\hbar^2 a_{\mathrm{sc}}/m$ and $\mathbf{p}_j = m\mathbf{v}_j$. Eq. (2a) describes the scattering of two thermal atoms with velocities $\mathbf{v}_3$ and $\mathbf{v}_4$ resulting in a BEC atom and a thermal atom with velocity $\mathbf{v}_2$; Eq. (2b) corresponds to the opposite process. In Eq. (2a) $\mathbf{v}_4 = \tilde{\mathbf{v}} + \frac{g n_{\mathrm{BEC}}}{m v_3^2}\mathbf{v}_3$ with $\tilde{\mathbf{v}} \perp \mathbf{v}_3$ such that the second integration is performed over the plane perpendicular to $\mathbf{v}_3$. In Eq. (2b) $\mathrm{d}\Omega$ denotes the solid angle subtended by $\mathbf{v}_3$ and $\mathbf{v}_4$. We use ^{84}Sr atomic mass m and its s-wave scattering length $a_{\mathrm{sc}} = 122.8\, a_0$, with a_0 the Bohr radius. $f_j = f(\mathbf{r}, \mathbf{p}_j)$ are the *unknown* distribution functions of the thermal atoms with the property $N_{\mathrm{th}} = \int \mathrm{d}\mathbf{r}\, \mathrm{d}\mathbf{p}/h^3 f(\mathbf{r}, \mathbf{p})$.

In principle, one could obtain f_j from complete N-body simulations[66] of the open system, accounting for the coupled dynamics between the reservoir, the dimple and the BEC. While strongly desirable, such a study goes beyond the scope of the present analysis[67]. Instead, let us first assume a thermal equilibrium such that f is given by the Bose-Einstein distribution. In this case the total number of non-interacting atoms in a harmonic trap is given by $N_{\mathrm{th}} = (\beta_D \hbar\omega_D)^{-3}\mathrm{Li}_3\left(\mathrm{e}^{-\beta_D\mu}\right)$. Here μ is the chemical potential of the distribution, $\omega_D = (\omega_{Dx}\omega_{Dy}\omega_{Dz})^{1/3}$, $\beta_D = 1/k_{\mathrm{B}}T_D$, and $\mathrm{Li}_s(z)$ is a polylogarithm of order s. With the experimentally measured dimple trap frequencies and gas temperature, we find that even for the maximum allowed $\mu = 0$, the atom number only reaches $N_{\mathrm{th}} \approx 1.8\times10^5$. This estimation is well below the measured value of $6.9(4)\times10^5$ atoms in the dimple. To try to resolve this discrepancy, we perform a more refined calculation following[68], where the harmonic trapping potential is replaced by the combined potential of the reservoir and dimple traps, and where we account for the interactions between atoms. This calculation gives $N_{\mathrm{th}} \approx 3.6\times10^5$ atoms in reservoir and dimple combined, which is less than those measured in the dimple, let alone in the reservoir and dimple combined $(1.4(2)\times10^6)$. Unsurprisingly, in this calculation most atoms are contained well within the dimple, as $k_{\mathrm{B}}T_D$ is much smaller than the dimple depth. The refined approach is thus also incapable of reproducing the measured atom number. This is in direct contradiction with our previous analysis in[68]. There, the refined calculation was capable of accounting for all atoms in a trap with a similar geometry, but where the continuous refilling of the reservoir was absent. This difference leads us to conclude that the current system does not fulfill the thermal equilibrium assumption due to its open character, and that the population dynamics is governed by an out-of-equilibrium distribution function f.

To proceed, we assume that the distribution function can be written as a product

$$f(\mathbf{r}, \mathbf{p}) = n_{\mathrm{th}}(\mathbf{r}) f'(\mathbf{p}). \tag{3}$$

We take $f'(\mathbf{p}) = \frac{1}{\mathcal{N}}\mathrm{e}^{-\beta_D\frac{p^2}{2m}}$, $\mathcal{N} = \frac{4\pi}{(2\pi\hbar)^3}\sqrt{\frac{\pi}{2}}\left(\frac{m}{\beta_D}\right)^{\frac{3}{2}}$ to be the Boltzmann distribution, which fulfills the normalization property $\int \frac{\mathrm{d}^3p}{(2\pi\hbar)^3} f'(\mathbf{p}) = 1$. We next specify the densities $n_{\mathrm{BEC}}(\mathbf{r})$ of the BEC and $n_{\mathrm{th}}(\mathbf{r})$ of the thermal atoms appearing in Eq. (1). For the BEC we consider the Thomas-Fermi profile given by

$$n_{\mathrm{BEC}}(\mathbf{r}) = n_0\left(1 - \left(\frac{x}{R_x}\right)^2 - \left(\frac{y}{R_y}\right)^2 - \left(\frac{z}{R_z}\right)^2\right), \tag{4}$$

where $R_\alpha = \sqrt{2\mu_D/m}/\omega_{D\alpha}$, $\mu_D = \frac{\hbar\omega_D}{2}\left(15 N_{\mathrm{BEC}}\frac{a_{\mathrm{sc}}}{a_{\mathrm{ho}}}\right)^{\frac{2}{5}}$, $a_{\mathrm{ho}} = \sqrt{\frac{\hbar}{m\omega_D}}$, N_{BEC} is the total BEC atom number and $n_0 = \mu_D/g$. In the BEC region, the condensate atoms will repel the thermal ones resulting, to a good accuracy,

in the characteristic parabolic profile

$$n_{\rm th}(\mathbf{r}) \approx n_{\rm th,0}\left[1+\gamma_x\left(\frac{x}{R_x}\right)^2+\gamma_y\left(\frac{y}{R_y}\right)^2+\gamma_z\left(\frac{z}{R_z}\right)^2\right], \tag{5}$$

where $\gamma_\alpha = (n_{c,\alpha} - n_{\rm th,0})/n_{\rm th,0}$.

Next, motivated by the approach in[66,68], we determine the thermal densities in the center, $n_{\rm th,0} = n_{\rm th}(0)$, and at the edge of the BEC cloud, $n_{c,\alpha} = n_{\rm th}(R_\alpha)$ in the direction $\alpha = x, y, z$, self-consistently as

$$n_{\rm th}(\mathbf{r}) = -\frac{1}{\lambda_{\rm dB}^3}{\rm Li}_{\frac{3}{2}}\left[-{\rm e}^{-\beta_D(V(\mathbf{r})+2gn_{\rm BEC}(\mathbf{r})+2gn_{\rm th}(\mathbf{r})-\eta)}\right], \tag{6}$$

with $\lambda_{\rm dB} = h/\sqrt{2\pi m k_B T_D}$ the thermal de-Broglie wavelength. Here, we need to stress that unlike in[68], the function in Eq. (6) is not the density corresponding to the thermal equilibrium of a Bose gas as that one fails to reproduce the observed number of thermal atoms $N_{\rm th}$, see the discussion above Eq. (3). The function in Eq. (6) in fact corresponds to a Fermi-Dirac distribution, which however here does not have a particular physical significance. It should be seen as a convenient ansatz from which we extract $n_{\rm th,0}$ and $n_{c,\alpha}$, which has the advantage that it allows to match the observed number $N_{\rm th}$ for positive values of the parameter η[69].

The assumed prescription for $n_{\rm th}(\mathbf{r})$ Eq. (6) is clearly an idealization. Despite this fact, the model yields a value for the three-body loss rate parameter γ_{3b} that is compatible with the values reported in the literature (see section *The results* below). We interpret this compatibility as indicating that the values of $n_{\rm th,0}$ and $n_{c,\alpha}$ extracted from Eq. (6) lie within a factor $O(1)$ from the actual experimental values.

Next, we turn to the evaluation of the collisional integrals Eqs. (2) using the distribution function Eq. (3) with the result

$$s_{\rm in} = n_{\rm BEC} n_{\rm th}^2 \sigma I_{\rm in} \tag{7a}$$

$$s_{\rm out} = n_{\rm BEC} n_{\rm th} \sigma I_{\rm out}^{(1)} + n_{\rm BEC} n_{\rm th}^2 \sigma I_{\rm out}^{(2)}. \tag{7b}$$

Here, we have introduced the functions

$$I_{\rm in} = \kappa v_0^2 K_1(\beta_D m v_0^2) \tag{8a}$$

$$I_{\rm out}^{(1)} = \kappa \mathcal{N} {\rm e}^{-\beta_D m v_0^2} v_0^2 K_1(\beta_D m v_0^2) \tag{8b}$$

$$I_{\rm out}^{(2)} = 2\kappa j, \tag{8c}$$

where $\kappa = \frac{1}{h^3}\frac{1}{\mathcal{N}^2}\frac{8\pi m^3}{m\beta_D}$, K_1 is the modified Bessel function of the second kind, and

$$j(\mathbf{r}) = \int_{2v_0}^{\infty} {\rm d}v_2\, v_2 {\rm e}^{-\frac{\beta_D m}{2}v_2^2}\left[{\rm e}^{-\frac{\beta_D m}{8}(v_2-\Delta v(v_2))^2} - {\rm e}^{-\frac{\beta_D m}{8}(v_2+\Delta v(v_2))^2}\right], \tag{9}$$

which has to be evaluated numerically.

Ultimately, we are interested in the dynamics of the total number of the condensate atoms, which can be obtained by integrating Eq. (1) over the volume. To be able to carry out the final integration, we take the functions $I_{\rm in}, I_{\rm out}^{(1,2)}$ to be independent of the position[70]. Using the profiles (4),(5) we arrive at the final rate equation

$$\dot{N}_{\rm BEC} = S_{\rm in} - S_{\rm out} - L_{\rm 1b} - L_{\rm 3b}^{(3)} - L_{\rm 3b}^{(2)} - L_{\rm 3b}^{(1)}, \tag{10}$$

where

$$S_{\rm in} = n_0 n_{\rm th,0}^2 \sigma V^{(1,2)} I_{\rm in} \tag{11a}$$

$$S_{\rm out} = n_0 n_{\rm th,0} \sigma V^{(1,1)} I_{\rm out}^{(1)} + n_0 n_{\rm th,0}^2 \sigma V^{(1,2)} I_{\rm out}^{(2)} \tag{11b}$$

$$L_{\rm 1b} = \gamma_{\rm 1b} N_{\rm BEC} \tag{11c}$$

$$L_{\rm 3b}^{(3)} = \gamma_{\rm 3b} n_0^3 V^{(3,0)} \tag{11d}$$

$$L_{\rm 3b}^{(2)} = 6\gamma_{\rm 3b} n_0^2 n_{\rm th,0} V^{(2,1)} \tag{11e}$$

$$L_{\rm 3b}^{(1)} = 6\gamma_{\rm 3b} n_0 n_{\rm th,0}^2 V^{(1,2)} \tag{11f}$$

In the above equations, we have introduced the volume integrals

$$V^{(p,q)} = 1/(n_0^p n_{\rm th,0}^q)\int {\rm d}\mathbf{r}\, n_{\rm BEC}(\mathbf{r})^p n_{\rm th}(\mathbf{r})^q, \tag{12}$$

which evaluate to

$$V^{(1,1)} = 4\pi R_x R_y R_z \frac{2}{105}(7+3\bar{\gamma}) \tag{13a}$$

$$V^{(1,2)} = 4\pi R_x R_y R_z \frac{2}{315}(21+5G+18\bar{\gamma}) \tag{13b}$$

$$V^{(2,1)} = 4\pi R_x R_y R_z \frac{8}{315}(3+\bar{\gamma}) \tag{13c}$$

$$V^{(3,0)} = 4\pi R_x R_y R_z \frac{16}{315}, \tag{13d}$$

where

$$\bar{\gamma} = \frac{1}{3}(\gamma_x+\gamma_y+\gamma_z) \tag{14a}$$

$$G = \frac{1}{5}(\gamma_x^2+\gamma_y^2+\gamma_z^2) + \frac{2}{15}(\gamma_x\gamma_y+\gamma_x\gamma_z+\gamma_y\gamma_z). \tag{14b}$$

To fit the data, we take $\gamma_{\rm 1b}, \gamma_{\rm 3b}$ in Eqs. (11c)-(11f) as free parameters and further parametrize Eq. (11a) and Eq. (11b) as

$$S_{\rm in} = \alpha_{\rm in} n_0 n_{\rm th,0}^2 \sigma V^{(1,2)} I_{\rm in} \tag{15a}$$

$$S_{\rm out} = \alpha_{\rm out}\alpha_{\rm in}(n_0 n_{\rm th,0}\sigma V^{(1,1)} I_{\rm out}^{(1)} + n_0 n_{\rm th,0}^2 \sigma V^{(1,2)} I_{\rm out}^{(2)}) \tag{15b}$$

with $\alpha_{\rm in}, \alpha_{\rm out}$ the fit parameters.

The results — We have simultaneously fitted 6 data sets of the onset of the BEC analogous to Fig. 3b, with slight variations in the experimental starting conditions. For each of these sets, we use as a known time-varying input our measurements of the time evolution of dimple atom number and temperature (see for example the exponential fits to the dimple data of Extended Data Fig. 7). We use the rate Eq. (10) and require the fit parameters $\gamma_{\rm 1b}$, $\gamma_{\rm 3b}$, and $\alpha_{\rm out}$ to be the same for all the data sets. Conversely, we allow independent variation for each data set of $\alpha_{\rm in}$ and the initial BEC atom number $N_{\rm BEC}(0)$, which provides the seed to Eq. (10).

From this single fit, we obtain the loss rate parameters $\gamma_{\rm 1b} = 6(2)\,{\rm s}^{-1}$ and $\gamma_{\rm 3b} = 1.9(2)\times 10^{-29}\,{\rm cm}^6/{\rm s}$. The value obtained for $\gamma_{\rm 3b}$ is compatible with the rough experimental values reported in the literature, namely $\approx 0.7\times 10^{-29}\,{\rm cm}^6/{\rm s}$ in[71] and $1.4(3)\times 10^{-29}\,{\rm cm}^6/{\rm s}$. The latter value can be extracted from the data used in[72], where the standard deviation is derived from statistical uncertainty, without accounting for systematic effects. We also obtain the average flux into the CW BEC at steady state $\bar{S}_{\rm in} - \bar{S}_{\rm out} = 2.4(5)\times 10^5\,{\rm atoms\,s}^{-1}$. In steady state, most of this incoming flux is compensated by the three-body losses involving thermal atoms described by Eqs. (11e) and (11f), while the loss mechanisms Eqs. (11c) and (11d) are only reaching a few $10^4\,{\rm atoms\,s}^{-1}$. Finally, we extract from the fit additional information about the CW BEC, namely the Thomas-Fermi radii $\{R_{\rm TFx}, R_{\rm TFy}, R_{\rm TFz}\} = \{2.8(1), 1.2(1), 2.9(1)\}\,\mu{\rm m}$, the peak BEC density $n_0 = 3.6(2)\times 10^{20}\,{\rm atoms\,m}^{-3}$ and the density of the thermal gas at the BEC center $n_{\rm th,0} = 3.6(1)\times 10^{20}\,{\rm atoms\,m}^{-3}$.

Extended Data Fig. 7 shows example fits together with their corresponding experimental data sets. The left ("loading" stage) of Extended Data Fig. 7 shows the onset and stabilization of the BEC following the switch on of the Zeeman slower beam. The right ("unloading" stage) of Extended Data Fig. 7 shows the subsequent data after suddenly switching off the same beam. This corresponds to stopping the atomic flux into the reservoir and leads to the disappearance of the BEC. The rate equation Eq. (10) captures well the initial dynamics of the BEC atom number as well as the initial dynamics of the unloading stage. The discrepancy for longer times during the unloading stage can likely be attributed to the change of the momentum distribution functions $f(\mathbf{r},\mathbf{p})$. This arises from the simultaneous thermalization and depletion of the dimple atoms, which is not captured by the parametrization of Eqs. (15).

In summary, the rate equation (10) provides a satisfactory fit to the data, from which we obtain the loss rate parameters $\gamma_{\rm 1b}$, $\gamma_{\rm 3b}$ and the steady-state BEC flux rate $\bar{S}_{\rm in} - \bar{S}_{\rm out}$. A more comprehensive quantitative understanding may be provided through further analysis starting from first principles. Such an analysis requires more complex modeling of the coupled dynamics between the reservoir, the dimple, and the BEC, such as the use of the ZNG theory[73,74] and the related N-body simulations[66].

---

[60] Ch. J. Bordé, "Amplification of atomic fields by stimulated emission of atoms," Phys. Lett. A **204**, 217 (1995).
[61] H.-J. Miesner, D. M. Stamper-Kurn, M. R. Andrews, D. S. Durfee, S. Inouye, and W. Ketterle, "Bosonic stimulation in the formation of a Bose-Einstein condensate," Science **279**, 1005 (1998).
[62] Mikio Kozuma, Yoichi Suzuki, Yoshio Torii, Toshiaki Sugiura, Takahiro Kuga, E. W. Hagley, and L. Deng, "Phase-coherent amplification of matter waves," Science **286**, 2309 (1999).
[63] S. Inouye, T. Pfau, S. Gupta, A. P. Chikkatur, A. Görlitz, D. E. Pritchard, and W. Ketterle, "Phase-coherent amplification of atomic matter waves," Nature **402**, 641 (1999).
[64] Y. Castin and R. Dum, "Bose-Einstein condensates in time dependent traps," Phys. Rev. Lett. **77**, 5315 (1996).
[65] Shovan Dutta and Erich J. Mueller, "Kinetics of Bose-Einstein condensation in a dimple potential," Phys. Rev. A **91**, 013601 (2015).
[66] B. Jackson and E. Zaremba, "Modeling Bose-Einstein condensed gases at finite temperatures with N-body simulations," Phys. Rev. A **66**, 033606 (2002).

[67] In this context, a possibility is to consider instead a simpler ergodic description of the Bose-Einstein condensation[75–77]. However, our preliminary analysis indicates breaking of the ergodicity assumptions, thus requiring the complete N-body simulations.

[68] Simon Stellmer, Benjamin Pasquiou, Rudolf Grimm, and Florian Schreck, "Laser cooling to quantum degeneracy," Phys. Rev. Lett. **110**, 263003 (2013).
[69] R. B. Dingle, "The Fermi-Dirac integrals," Appl. Sci. Res. **6**, 225 (1957).

[70] In principle, they still depend on spatial coordinates through $v_0(n_{\rm BEC}(\mathbf{r}))$. For the span of experimental values of $n_{\rm BEC}(\mathbf{r})$, this leads to a maximum variation by a factor 0.7.

[71] Yenny Natali Martinez de Escobar, Bose-Einstein condensation of ^{84}Sr, Ph.D. thesis, Rice University (2012).
[72] Simon Stellmer, Rudolf Grimm, and Florian Schreck, "Production of quantum-degenerate strontium gases," Phys. Rev. A **87**, 013611 (2013).
[73] E. Zaremba, T. Nikuni, and A. Griffin, "Dynamics of trapped Bose gases at finite temperatures," J. Low Temp. Phys. **116**, 277 (1999).
[74] M. J. Bijlsma, E. Zaremba, and H. T. C. Stoof, "Condensate growth in trapped Bose gases," Phys. Rev. A **62**, 063609 (2000).
[75] O. J. Luiten, M. W. Reynolds, and J. T. M. Walraven, "Kinetic theory of the evaporative cooling of a trapped gas," Phys. Rev. A **53**, 381 (1996).
[76] C. W. Gardiner, M. D. Lee, R. J. Ballagh, M. J. Davis, and P. Zoller, "Quantum kinetic theory of condensate growth: Comparison of experiment and theory," Phys. Rev. Lett. **81**, 5266 (1998).
[77] M. Holland, J. Williams, and J. Cooper, "Bose-Einstein condensation: Kinetic evolution obtained from simulated

trajectories," Phys. Rev. A **55**, 3670 (1997).
